# Designing Enzymatic Reactivity with an Expanded Palette

**DOI:** 10.1002/cbic.202500076

**Published:** 2025-04-04

**Authors:** Reuben B. Leveson‐Gower

**Affiliations:** ^1^ Biocatalysis Section Department of Biotechnology Delft University of Technology Van der Maasweg 9 2629HZ Delft The Netherlands

**Keywords:** biocatalysis, directed evolution, noncanonical amino acids, photobiocatalysis, unnatural cofactors

## Abstract

The expanding applications of biocatalysis in the chemical and pharmaceutical sectors herald a greener future for these industries. Yet, the range of chemical reactions known to enzymes only covers a small fraction of what is required for modern synthetic routes. To continue the increases in sustainability afforded by converting chemical processes into enzymatic ones, fundamentally new kinds of biocatalytic reactivity are required. Perhaps the very components from which enzymes are constructed, a palette of canonical amino acids and cofactors, inherently limit their catalytic possibilities, even if all the available natural sequence space can be explored. In recent years, there has been an explosion of strategies to produce new biocatalytic function through the incorporation of noncanonical amino acids and synthetic cofactors, new colors which are added to the enzyme design palette. This has enabled new enzymatic reactions that proceed via organocatalytic, organometallic, and photocatalytic mechanisms. Aside from designing new enzymatic activities from scratch, exogenous photocatalysts have recently also been used in synergy with natural enzyme active sites to diverge their reactivity towards radical pathways. This review will highlight recent developments in enriching enzymatic chemistry with new unnatural components, providing an outlook for future directions and needed developments for practicality and sustainability.

## Graphical abstract

Innovation in biocatalysis is rapidly increasingly the diversity of catalytic reactivity that can be mediated by enzymes, addressing a key bottleneck for their widespread adoption in industrial chemical synthesis. A key approach to this is building enzymes with unnatural catalytic components that provide an expanded palette with new possibilities for enzymatic reactivity.

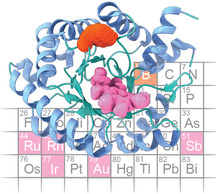

## 1 Introduction

**Figure 1:**
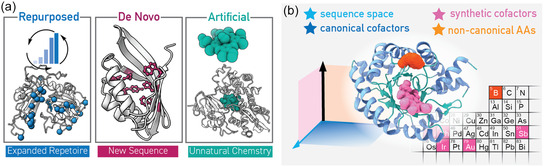
Approaches for the expansion of enzyme function. a) Three general methods have been demonstrated to create new enzymatic catalysis—repurposing of natural or engineered enzymes via directed evolution; design of de novo sequences from scratch with novel active‐site amino acid constellations; addition of unnatural components (e.g., to produce artificial enzymes). b) Expansion of available chemical space beyond the limitations of amino acid sequence space and canonical cofactors to include synthetic cofactors and ncAAs creates opportunities for new types of reactivity, and even the use of “nonbiological” elements. Highlighted on the period table segment pictured are elements which are not known to be utilized by natural enzymes for catalysis, but which have been used to generate artificial enzymes through the use of synthetic cofactors (pink) or ncAAs (orange).

Although a few classes of enzymes are nowadays regularly applied for even large‐scale synthetic processes in the pharmaceutical industry, the number of types of chemical steps that are currently addressed with an enzyme in industrial synthesis remains rather limited.^[^ ^1^, ^2^, ^3^ ^]^ That said, the examples where enzymes are applied for synthesis in industry do provide real benefits from the use of a biocatalytic process.^[^ ^4^, ^5^, ^6^, ^7^, ^8^ ^]^ Process mass intensity can often be greatly reduced, and product purity greatly enhanced, leading to the associated financial incentives that are ultimately required for commercial adoption of any technology change.^[^ ^9^, ^10^, ^11^, ^12^, ^13^, ^14^, ^15^ ^]^ Therefore, the expansion of biocatalytic reactivities to include more known chemistry (an area which has been referred to as “chemomimetic biocatalysis”^[^ ^16^, ^17^ ^]^) deserves urgent developments since real potential for disruptive innovation in the chemical industry clearly exists.^[^ ^18^ ^]^ To achieve this diversification, several complementary approaches have been explored (**Figure** **1**a).

**(I) Enzyme repurposing** through directed evolution is a powerful way to realize new chemical reactivities.^[^ ^19^, ^20^ ^]^ Most well known, perhaps, are the examples of unnatural chemistry in cytochrome P450, other heme‐containing enzymes, and noncatalytic proteins pioneered by Frances Arnold, where chemistry proceeds via reactive intermediates isolobal to the native Fe^IV^‐oxo species “Compound I.”^[^ ^21^ ^]^ Many more examples of similar tactics for rewiring the mechanisms of diverse enzyme classes can be found in the literature.^[^ ^22^, ^23^ ^]^ However, directed evolution campaigns require at least a measurable trace activity, which may not always be possible to identify starting from panels of natural or engineered enzymes.

**(II) De novo enzyme design** provides the opportunity to escape the sequence space available to natural or engineered enzymes by inserting designed active site amino acid side‐chain constellations into existing protein folds, or even completely unnatural sequences.^[^ ^24^, ^25^, ^26^, ^27^, ^28^, ^29^ ^]^ Achieving the design accuracy necessary to choreograph a sequential catalytic reaction (while likely necessitates “multistate design”^[^ ^30^, ^31^, ^32^, ^33^ ^]^) is a formidable challenge and, while newer AI‐based methods are beginning to address this;^[^ ^27^ ^]^ efforts toward generalizable strategies to realize highly efficient de novo enzymes without follow‐up empirical optimization via directed evolution^[^ ^26^, ^34^ ^]^ are still ongoing.^[^ ^28^, ^29^ ^]^

**(III) Artificial enzymes,** hybrid constructions of synthetic and proteogenic components, give the possibility to take advantage of chemistries that have not been realized with natural enzymes’ catalytic machinery or that are chemically impossible with only natural proteogenic components (i.e., amino acids, metal ions, and cofactors).^[^ ^35^, ^36^, ^37^, ^38^ ^]^

Incorporation of these unnatural components in the enzyme design process opens a new dimension of chemical space, moving beyond the restrictions to which nature has been subject and taking advantage of the wealth of catalysis knowledge accumulated by chemists (Figure 1b). Through addition of new unnatural components in enzymatic systems, the available palette for enzyme design (amino acids, cofactors) is expanded to include new colors, which can help create a richer catalytic language with increased possibilities. It should be noted that the three categories identified here also overlap. Artificial enzymes commonly must undergo directed evolution to achieve better performance or to be repurposed for other catalytic applications, and computational enzyme design protocols can also be adapted for use with unnatural components. Comprehensive reviews are available on the topics of artificial metalloenzymes and de novo enzyme design.^[^ ^24^, ^25^, ^35^, ^39^, ^40^, ^41^ ^]^ This review will focus on the most significant recent contributions to, and themes in, the design of unnatural enzymes with metal‐containing, organic, and main‐group catalytic components from 2020 onward. This review will also survey the recent application of unnatural catalytic components (photocatalysts) for synergy with natural enzymatic chemistry for new reaction outcomes. Finally, I provide my perspectives on the frontier challenges for ultimate practicality and sustainability of this research area, to facilitate genetic optimization and reduce catalyst costs.

## 2 Incorporating Unnatural Components

This review starts with a brief description of the technical strategies that have been employed to add new colors to enzyme designer's palette. These strategies will form the basis for the rest of the review, and will be signaled with their respective icons in the following figures.

**Figure 2:**
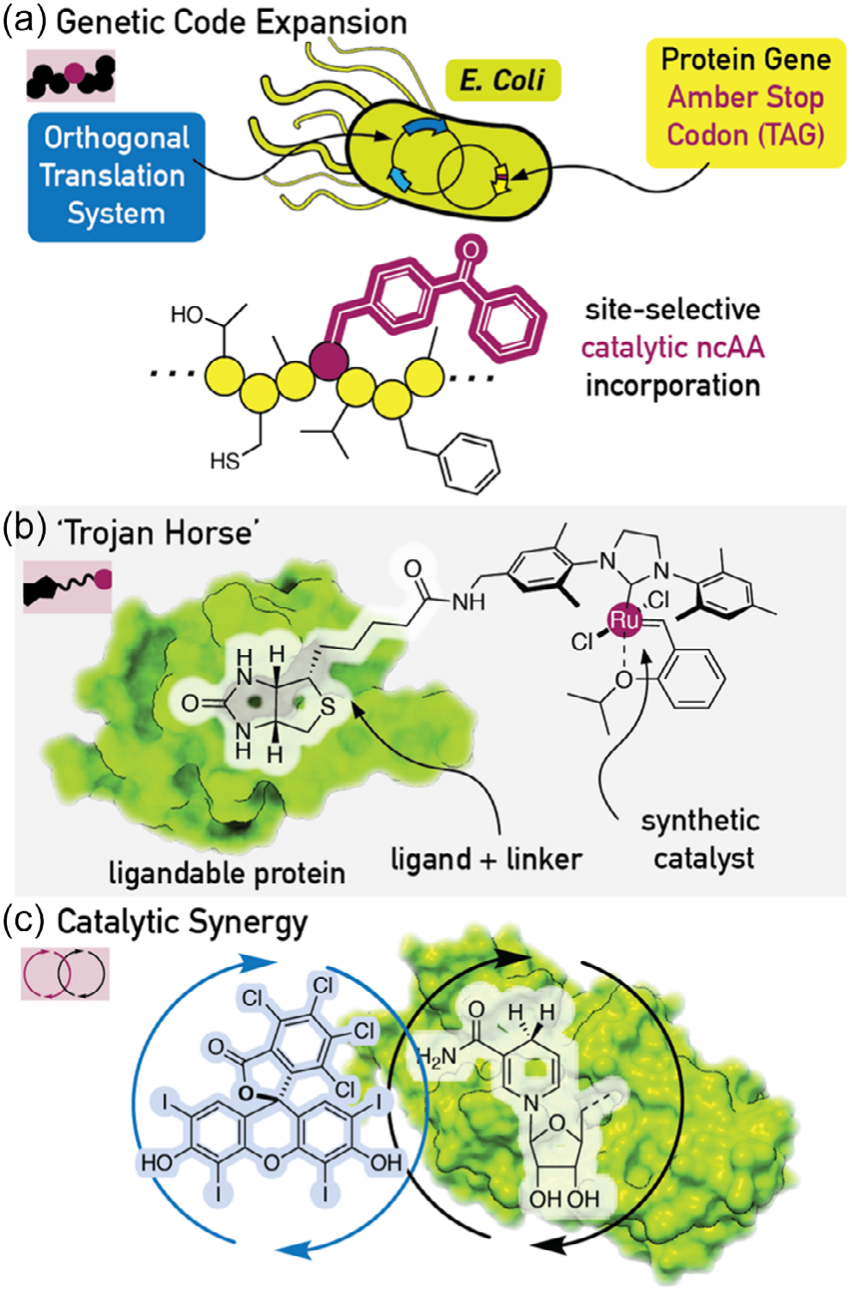
Methods for linking unnatural components to enzymes. a) Genetic code expansion—the incorporation of ncAAs via amber stop codon (TAG) suppression enabled by OTSs. b) The “Trojan Horse” method where the affinity of a protein for (analogues of) its natural ligand is exploited to bind synthetic catalysts. c) Catalytic synergy, where an unnatural catalyst cooperates with a natural enzyme catalytic center to enable a new mechanistic manifold.

**(I) Genetic code expansion** (**Figure** **2**a) enables the direct incorporation of new chemical functionalities in the form of unnatural side chains afforded by noncanonical amino acids (ncAAs) which are condensed directly into the protein polyamide backbone.^[^ ^42^ ^]^ The technique of amber (nucleotides TAG) stop codon suppression (SCS) is the most widely used and, so far, convenient method to achieve this. A specialized amino‐acyl tRNA synthetase/tRNA pair (engineered through directed evolution) serves as an orthogonal translation system (OTS).^[^ ^43^ ^]^ For example, the OTS can be assembled on a single plasmid, while another plasmid encodes the targeted protein for ncAA incorporation with a TAG codon in the intended position for ncAA incorporation. Hundreds of ncAAs have been incorporated using SCS, with applications in diverse applications from chemical biology to biocatalysis.^[^ ^36^, ^37^, ^44^, ^45^, ^46^ ^]^ Inevitably, some portion of the amber stop codon is effective, giving truncate protein which ultimately reduces target protein yield. The ratio of truncated to full‐length protein depends on the overall efficiency of the OTS, which is not always clearly reported in the literature. While the number of ncAAs demonstrated for incorporation is large, even with extensive engineering, the substrate scope of aaRSs is still limited with respect to both steric and electronic properties of the targeted sidechain.

**(II) The “Trojan horse” strategy** (Figure 2b) exploits the affinity of a protein scaffold, for example, for a native ligand (e.g., biotin for streptavidin,^[^ ^47^ ^]^ a sulfonamide for carbonic anhydrase,^[^ ^48^ ^]^ or a siderophore for a periplasmic binding protein^[^ ^49^ ^]^) to bind an unnatural catalytic group in the protein pocket through construction of a ligand‐catalyst conjugate by chemical synthesis. Typically, a flexible linker region is employed between the catalyst and ligand which, in the case of streptavidin, results in a catalyst center that is not well buried in the protein structure, making effective protein engineering challenging. While this strategy can give complete control over the nature of the synthetic catalyst employed, controlling the active site environment can be challenging, and the cofactor can present a further challenge in its synthesis. In some cases, a protein scaffold displays promiscuous binding of a synthetic catalyst without the need to link it to a known ligand. For example, the multidrug resistance regulatory protein LmrR binds a variety of planar metal complexes,^[^ ^50^ ^]^ and several heme‐binding proteins accommodate synthetic porphyrin complexes with altered backbones or swapped metal centers.^[^ ^51^ ^]^ Here, extensive structural differences from the natural ligand(s) will likely result in significantly reduced affinities and pose challenges in ensuring selective catalysis. In the case of unnatural metal‐porphyrin derivatives in hemoproteins, dissociation constants are commonly in the micromolar range which is more similar to heme‐binding proteins involved in heme‐shuttling rather than in heme‐containing enzymes.^[^ ^52^, ^53^ ^]^

**(III) Synergistic catalysis** (Figure 2c) entails the cooperation between a reactive intermediate of a synthetic catalyst and a reactive intermediate in an enzyme's native active site to produce a novel mechanistic outcome by merging two catalytic cycles. To date, synthetic photocatalysts have been used in this manner to unlock new mechanistic pathways in a variety of different enzyme active sites (see Section 5). Here, the association between the synthetic catalyst and enzyme is not precisely controlled, but rather different catalysts can be screened empirically. This strategy is certainly pragmatic, using off‐the‐shelf chemicals, but could lead to, e.g., selectivity issues through nonspecific catalyst assembly.

Combined, these strategies provide an expanded palette for enzyme designers to generate novel catalytic activities in enzymes, as will be clearly demonstrated throughout this review. However, as noted, these strategies have their particular strengths and weaknesses which may need to be addressed for practical application and will be further discussed in the outlook section.

## 3 New Metal‐Centered Biocatalysis

Homogenous and organometallic catalysis are mainstays in modern organic and industrial chemistry, enabling a broad array of synthetic transformations from research laboratories to full‐scale chemical plants.^[^ ^54^, ^55^, ^56^ ^]^ The awards of the Nobel Prizes in Chemistry in 2005 and 2010 for the development of transition‐metal catalyzed metathesis and cross‐coupling reactions, respectively, are testament to the crucial role such methodologies now play. Correspondingly, in the past two decades that artificial enzymes have been intensively developed, the major research theme has been to translate unnatural metal‐centered mechanisms into enzymes, and in the last few years the frontiers in this topic are still being pushed forward.

### 3.1 New Metal‐Containing Active Sites through the “Trojan Horse” Strategy

**Figure 3:**
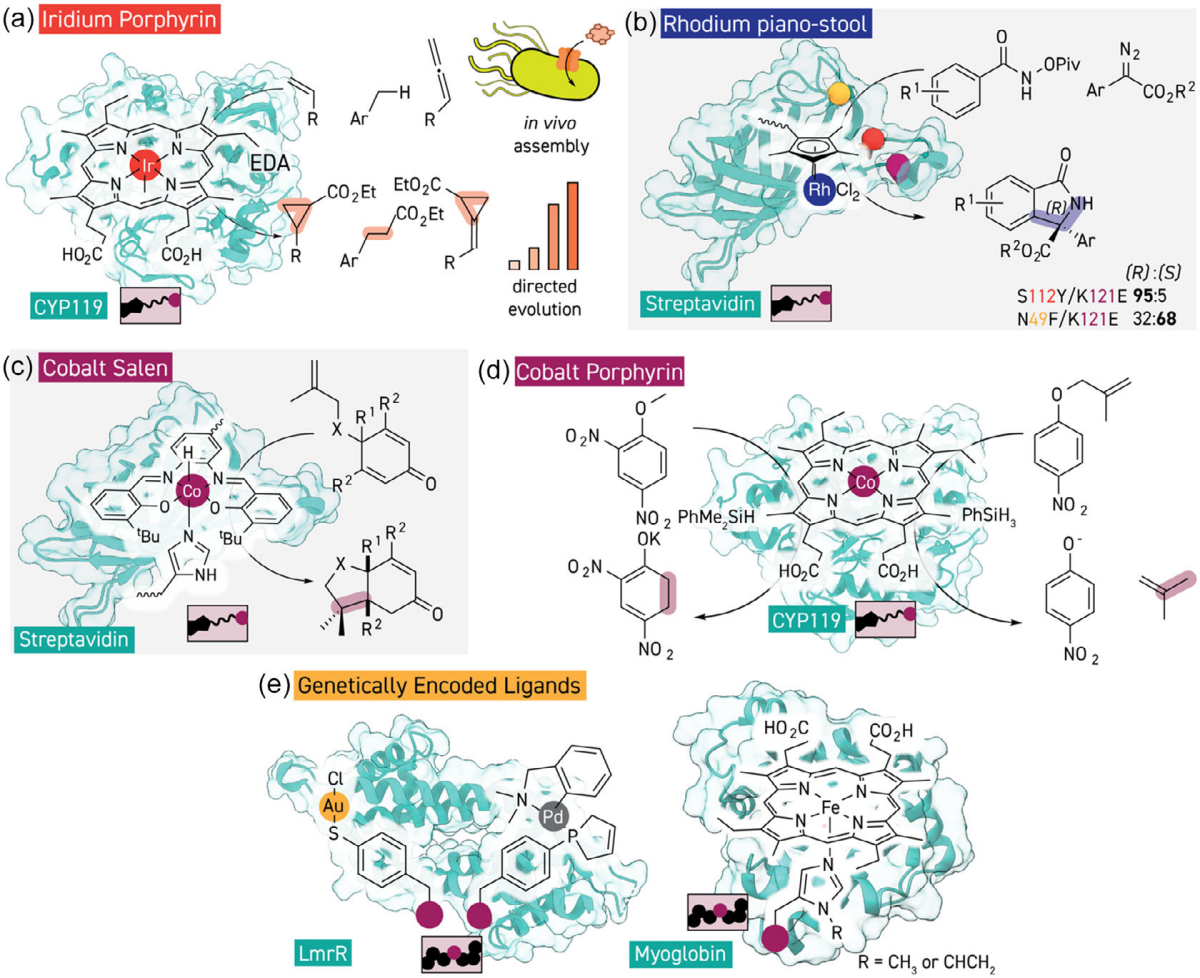
Addition of unnatural metal‐containing cofactors to protein scaffolds to generate enzymes with novel reactivities. a) Incorporation of an iridium‐porphyrin into cytochrome CYP119 affords an active and selective artificial enzyme for carbene transfer reactivities which can be assembled in whole cells for directed evolution and in vivo catalysis (PDB 1IO7). b) Incorporation of a rhodium piano‐stool complex into streptavidin by conjugation to biotin creates an artificial enzyme for isoindolone formation, position of the engineered residues 49, 112, and 121 shown in yellow, orange and crimson, respectively (PDB 1STP). c) Incorporation of a cobalt–salen complex by the same methodology creates a radical cyclase (PDB 1STP). d) Hydrogen atom transfer chemistry with cobalt‐substituted hemoprotein biosynthetically produced in *E. coli* (PDB 1IO7). e) Noncanonical amino acids are being used to create unnatural metal binding sites in proteins and influence the coordination environment and reactivity of natural metalloprotein active sites (PDB 6I8N and 6M8F).

The replacement of the heme‐cofactor in hemoproteins can be readily achieved by first removing the heme by treatment with acid, which ejects the heme from the active site (by protonation of the propionate groups^[^ ^57^ ^]^) into the bulk solvent. Subsequent extraction of the heme with 2‐butanone from the aqueous solution into the organic layer^[^ ^58^ ^]^ generates a solution of the apoprotein which can then be neutralized for reconstitution with a variety of synthetic cofactors with modified ligand frameworks or alternative metals, which was demonstrated in cytochrome c peroxidase as early as the 1960s.^[^ ^51^, ^59^, ^60^ ^]^ This technique has been broadly applied for mechanistic investigations of hemoproteins to achieve precise chemical editing of the cofactor.^[^ ^61^ ^]^ Fasan, Hayashi, Hartwig, and others have employed cofactor replacement to modulate and expand the catalytic reactivity of hemoproteins with synthetic cofactors containing metal‐substitutions and backbone modifications.^[^ ^62^, ^63^, ^64^, ^65^, ^66^, ^67^, ^68^, ^69^, ^70^, ^71^ ^]^ A significant portion of developments in this topic since 2020 has come from Hartwig's group, who have extensively explored the catalytic capabilities of cytochrome CYP119 from the thermophilic archaeon *Sulfolobus acidocaldarius*, reconstituted with methyliridium(III) mesoporphyrin (Ir(Me)‐MPIX), for carbene and nitrene transfer chemistry (**Figure** **3**a). Heterologous overexpression of CYP119 (but also P450‐BM3 and myoglobin) in *E* *scherichia* *coli* BL21‐DE3 under conditions which minimize heme biosynthesis using M9 minimal medium affords the apoprotein which can be reconstituted without the need for purification, facilitating high‐throughput experimentation and hence directed evolution.^[^ ^62^, ^63^, ^72^ ^]^ To obviate the need for cell lysis and achieve in vivo assembly of the artificial enzyme, the coexpression of heme‐transporting ChuA transporter or hug operon was demonstrated to permit the promiscuous transport of Ir(Me)‐MPIX into *E. coli* cells.^[^ ^73^, ^74^ ^]^ In this way, the carbene transfer reactivity of the artificial enzyme could be interfaced with a heterologously expressed biosynthetic pathway which produces limonene from glucose to afford unnatural terpenoids in a stereoselective fashion.^[^ ^73^ ^]^ Kinetic and crystallographic investigations of this artificial enzyme suggests that a dynamic flip of the unnatural cofactor in the enzyme active site may be responsible for catalyst activation.^[^ ^53^ ^]^ Using the same *E. coli* system, a directed evolution campaign was conducted in whole cells to identify mildly enantioselective mutants for a carbene N—H insertion reaction.^[^ ^74^ ^]^ In this carbene insertion to *N*‐alkyl aniline N—H bonds, the ylide intermediate produced after N—C bond formation may dissociate from the metal center and thereafter form the stereocenter by protonation, meaning that in both chemical and enzymatic approaches significant enantioselectivity was long elusive.^[^ ^75^ ^]^ In whole cells enantioselectivity could be observed thanks to selective assembly of the artificial enzyme by active transport of the unnatural cofactor, permitting a directed evolution campaign that produced a moderately enantioselective mutant. Most recently, allenes were employed as substrates for a carbene transfer reaction where directed evolution provided variants with divergent stereochemical reaction outcomes.^[^ ^76^ ^]^ Molecular dynamics simulations suggested that the mutations introduced biased the conformation of the iridium carbene intermediate to afford the stereoselective reaction pathways. It should be noted that carbene transfer to C—H, C=C, and N—H bonds has also been widely reported with engineered hemoproteins containing their natural cofactor.^[^ ^77^, ^78^, ^79^ ^]^ In some cases, iridium‐porphyrin reconstituted hemoproteins do provide higher turnover numbers for carbene transfer reactions than their iron‐containing counterparts, albeit on different substrates, yet it is currently unclear whether this truly justifies the use of this rare metal.^[^ ^63^ ^]^

Pioneered by Whitesides,^[^ ^47^ ^]^ but extensively elaborated over the last two decades by Ward,^[^ ^80^ ^]^ the (strept)avidin–biotin technology has been utilized to create artificial enzymes using unnatural biotin‐linked cofactors encoding a broad array of different chemical reactivities, with metal‐containing cofactors featuring heavily in the works to date. The S112 and K121 positions have been systematically explored for their influence on the performance of ruthenium‐ and gold‐dependent artificial streptavidin‐based enzymes by screening all 400 possible double mutants with various synthetic cofactors and substrates by Jeschek and Ward (position of these residues in the streptavidin monomer is shown in Figure 3b).^[^ ^81^ ^]^ By screening this library with different unnatural cofactors for several target activities, specialized mutants which favor each cofactor/reaction combination could be identified. This approach was further expanded to 20 residues in the secondary coordination sphere of the synthetic cofactor including multiple‐site mutants, using a relatively affordable yet precise library construction method based on oligonucleotide pools.^[^ ^82^ ^]^ Working in 96‐well microtiter plates, well‐ and plate‐specific barcoding for next generation sequencing allowed comprehensive construction of a sequence/activity dataset with over 2000 individual points that was used to train a machine learning algorithm which suggested a follow‐up library with a significantly increased hit rate. Other recent developments include an artificial enzyme with an iridium cofactor based on the streptavidin scaffold which can catalyze an intramolecular nitrene insertion into unactivated C—H bonds,^[^ ^83^ ^]^ seemingly providing a unique advantage over engineered myoglobin variants lacking the noble metal which can perform this reaction only on benzylic C—H bonds.^[^ ^84^ ^]^ The Maiti group recently demonstrated that streptavidin equipped with a rhodium piano‐stool complex via this technology could catalyze the formation of isoindolines by subsequent C—H insertion and reductive elimination.^[^ ^85^ ^]^ Mutations at the well‐explored S112 and K121 positions, proximal to the cofactor binding site, boosted activity and selectivity. Crystallographic characterization of the artificial enzyme facilitated production of enantiodivergent variants by rational engineering at the previously underexplored N49 residue, which is also adjacent to the synthetic cofactor (Figure 3b).

Moving away from late‐transition metals toward first‐row transition metals, Ward described the construction and engineering of a cobalt based artificial enzyme with a salen‐ligand framework in the streptavidin scaffold that catalyzes a radical cyclization reaction that proceeds via metal‐hydride hydrogen atom transfer (MHAT) (Figure 3c).^[^ ^86^ ^]^ Building on previous work demonstrating the insertion of loops to increase shielding around the relatively solvent‐exposed active‐site,^[^ ^87^ ^]^ chimeric streptavidin variants were computationally designed to buttress the active site and provide steric shielding to boost enantioselectivity. Buller and co‐workers demonstrated completely biosynthetic production of a cobalt‐substituted CYP119 variant which could also be employed for hydrogen atom transfer chemistry (Figure 3d).^[^ ^88^ ^]^ Compared to production of the native iron‐containing variants, supplementation of the medium with cobalt was the only extra required step, although protein titers were modest. UV–vis spectroscopy evidenced the formation of a putative cobalt‐hydride intermediate in the presence of phenylsilane, a reactive intermediate not accessible to iron‐porphyrins. Transfer of this hydrogen atom to *para*‐nitrophenol (PNP) allyl‐ethers releases the PNP leaving group allowing spectrometric detection of the reactivity, along with formation of a butene by‐product. This analysis method allowed screening of a large combinatorial active site mutant library, identifying a quadruple mutant with improved activity. Unexpected desaturative dearomatization reactivity was identified as the cause of noncharacteristic color changes observed with some substrates in the presence of this improved variant, and, in the presence of dimethylphenylsilane, application of a prochiral dinitrophenol derivative afforded the corresponding cyclohexadiene product with modest enantioselectivity. Two recent studies by the Ward and Chen groups have shown the utility of heme and nonheme iron enzymes for MHAT chemistry achieving radical cyclization and alkene hydration reactions respectively.^[^ ^89^, ^90^ ^]^ Further developments in enzymatic MHAT reactivity will likely come from both engineered natural enzymes, as well as those constructed with unnatural components.

### 3.2 Genetically Encoded Ligands for Metalloenzyme Design

Genetic code expansion has played an important role in the creation of unnatural metalloproteins^[^ ^91^ ^]^ as well as artificial enzymes and to modulate the coordination environment of natural metalloenzymes.^[^ ^36^, ^37^, ^92^ ^]^ Initial reports from Roelfes demonstrated the utility of the ncAAs 2,2′‐bipyridine alanine and hydroxyquinoline alanine in the creation of Cu^2+^‐based artificial enzymes operating via Lewis acid catalysis.^[^ ^93^, ^94^, ^95^ ^]^ More recently, an ncAA with a thiophenol side chain was employed by Roelfes and co‐workers to recruit a variety of late‐transition metals into the hydrophobic core of the LmrR protein scaffold (Figure 3e).^[^ ^96^ ^]^ By utilizing a previously described polyspecific OTS,^[^ ^97^ ^]^ selective incorporation of the ncAA was achieved, and its binding to gold created an artificial enzyme for a hydroamination reaction whose activity could be further optimized by site‐directed mutagenesis. A phosphine containing ncAA has also now been added to the collection of ncAAs for designing metalloproteins, although it must be incorporated in a borane‐protected form to prevent oxidation.^[^ ^98^ ^]^ Removal of the borane to allow metal binding after incorporation by amber stop codon suppression proved challenging, although near‐quantitative binding of a palladium complex was achieved after several days of incubation. However, the resulting unnatural metalloprotein has not been applied for catalysis yet. In 2003, Lu demonstrated that substitution of the axial methionine in azurin with isostructural ncAAs could be used to modulate the reduction potential.^[^ ^99^ ^]^ Hilvert, Fasan, and Green have extensively explored the role of substitutions of the axial ligand of myoglobin with ncAAs on its promiscuous carbene‐transfer, nitrene‐transfer, and peroxidase catalysis.^[^ ^100^, ^101^, ^102^, ^103^ ^]^ Here, the axial ligand substitution can boost both catalytic activity and oxygen tolerance of the resulting artificial enzymes compared to the natural histidine residue. Explored substitutions include methylation of the noncoordinating nitrogen atom and vinylation which was demonstrated by the group of Jie Wang,^[^ ^104^ ^]^ as well as switching the heteroaromatic moiety from imidazole to thiazole, thiophene, and pyridine. ncAAs provide the ability for precise chemical editing of active site of metalloenzymes and have also now been used to modulate reactivity in nonheme iron enzymes.^[^ ^105^ ^]^

## 4 Main‐Group and Organocatalytic Mechanisms

Organocatalysis, the use of nonmetal‐containing small molecules as catalysts, has also had a profound impact on asymmetric synthetic strategies in academia and industry, particularly in this century where the majority of the seminal work of 2021 Chemistry Nobel Laureates List and MacMillan has taken place.^[^ ^106^, ^107^ ^]^ The renaissance in organocatalysis in the late 1990s was inspired by natural enzymatic systems which employ only amino acid side chains to form their active site and operate via covalent mechanisms (e.g., Class I Aldolases which employs a catalytic lysine for enamine formation).^[^ ^108^ ^]^ The realization of small molecule analogues of these enzymatic systems (i.e., catalytic amines) allowed the systematic expansion of the available catalytic mechanisms yet, while many of these reactions are scalable, high catalyst loadings are often required. Enzymes which operate by organocatalytic mechanisms can be applied in efficient synthesis^[^ ^109^ ^]^ and are amenable to directed evolution to elaborate unnatural mechanistic pathways.^[^ ^110^ ^]^ The active site architecture of such enzymes (e.g., Class I Aldolase) necessitates the cooperation of several precisely placed side chains, and thus design of such active sites from scratch in order to reset the biases of natural enzyme scaffolds for particular mechanisms or substrates is a formidable (though tractable^[^ ^111^, ^112^ ^]^) challenge. Accordingly, many research efforts in recently years have been focused on equipping protein scaffolds with catalytic motifs inspired by small molecule organocatalysts to realize the benefits of both biocatalytic efficiency (by directed evolution) and mechanistic/substrate plasticity.

### 4.1 Aminocatalytic Artificial Enzymes

**Figure 4:**
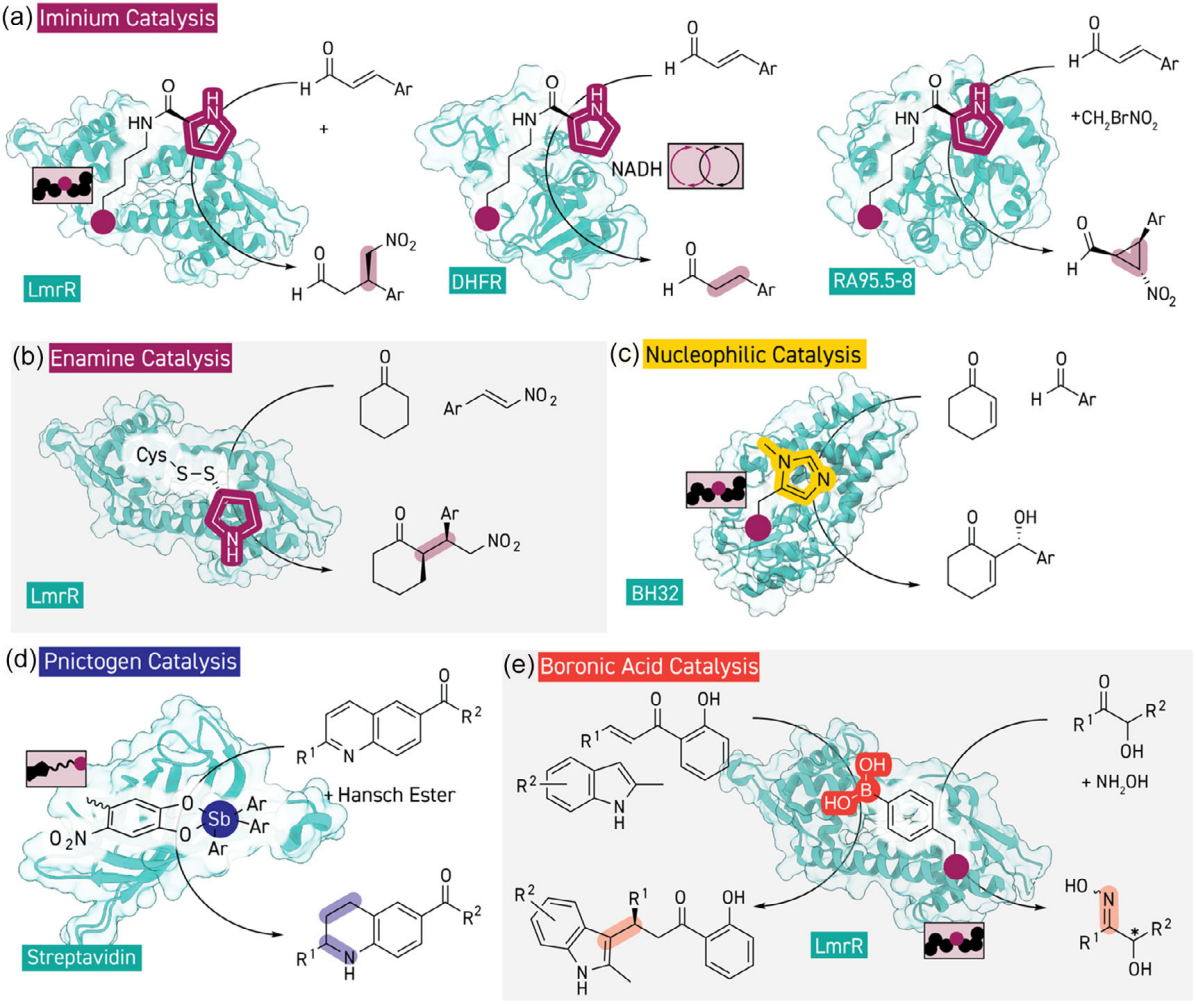
Main‐group and organocatalytic and strategies for enzyme design with unnatural components. a) Genetically encoding iminium catalysis in various scaffold proteins with an ncAA containing a proline‐sidechain enabled biocatalytic Michael additions, reductions, and cyclopropanations (PDB 6I8N, 1RA1, and 8XYN). b) Disulfide mediated assembly of an LmrR‐based artificial enzyme with unnatural pyrrolidine moiety which catalyzes Michael additions via an enamine intermediate (PRB 6I8N). c) The ncAA *N*‐methyl histidine acts as catalytic nucleophilic residue to enable a Morita–Baylis–Hillman reaction (PDB 8BP0). d) An antimony‐based unnatural cofactor anchored with the streptavidin–biotin technology affords 1,4‐reduction of a quinoline substrate in the presence of a Hansch ester cosubstrate (PDB 1STP). e) Boronic‐acid catalysis in the LmrR‐scaffold enabled by the ncAA pBoF, which enables oxime formation and Friedel–Crafts alkylation (PRB 6I8N).

Catalytic (secondary and primary) amines are the most well studied organocatalysts, and several examples of the incorporation of unnatural aminocatalytic components into protein scaffolds to create organocatalytic artificial enzymes can be found in the literature. This approach was originally realized for the iminium catalysis mechanism^[^ ^106^ ^]^ by Roelfes using the ncAA *para*‐aminophenylalanine (pAF) which bears an aniline group as its sidechain and can promote catalysis via iminium intermediates for hydrazone and oxime formation, as well as Friedel–Crafts alkylation reactions.^[^ ^113^, ^114^, ^115^, ^116^ ^]^ Most recently, the same group demonstrated that switching pAF for 3‐aminotyrosine inverts the enantioselectivity of the Friedel–Crafts vinylogous alkylation of indoles.^[^ ^117^ ^]^ Aromatic amines are less well represented among organocatalysts and exhibit particular substrate limitations (converting aldehydes but rarely ketones^[^ ^118^, ^119^ ^]^), but now ncAAs bearing pyrrolidine‐containing side chains, a widely used organocatalyst structure, have also been demonstrated for use as unnatural catalytic residues (**Figure** **4**a). The dipeptide formed from d‐proline and l‐lysine has proven to be a very versatile catalytic residue, and when incorporated in the widely used artificial enzyme scaffold protein LmrR it can promote a Michael addition of nitromethane to cinnamaldehyde via an iminium intermediate.^[^ ^120^, ^121^ ^]^ This reaction has also been explored extensively with the natural promiscuous enzyme 4‐oxalocrotonate tautomerase (4‐OT) and more recent with a class I aldolase, demonstrating that unnatural components are not necessary for this reactivity.^[^ ^110^, ^122^ ^]^ The same design strategy has proven applicable to a variety of other protein scaffolds, suggesting that this ncAA provides a catalytic potency that can function in several protein microenvironments without direct dependency on the cooperation of other specific side chains This catalytic ncAA can also synergistically cooperate with the native cofactor of dihydrofolate reductase to achieve the chemo‐ and stereo‐selective reduction of (beta‐substituted) cinnamaldehyde derivatives via iminium catalysis.^[^ ^123^ ^]^ Xiang and co‐workers incorporated this amino acid into an evolved computationally designed aldolase, after substituting the catalytic lysine residues for other amino acids giving an inactive enzyme, to produce an artificial enzyme for a nitrocyclopropanation reaction of cinnamaldehyde derivatives that proceeds via a tandem iminium/enamine mechanism.^[^ ^124^ ^]^ Interestingly, the original evolved/designed aldolase that was applied as scaffold protein can also catalyze the reaction using its designed active site of only natural amino acids, albeit with lower selectivity, and a similar cyclopropanation reaction has been demonstrated with 4‐OT.^[^ ^125^ ^]^ The streptavidin–biotin technology has also been recently applied to design artificial enzymes for iminium catalysis with pyrrolidine‐based cofactors.^[^ ^126^, ^127^, ^128^ ^]^

Enamine catalysis is the other main methodology of amine catalysis,^[^ ^107^ ^]^ and has been demonstrated with the enzymes 4‐OT^[^ ^129^, ^130^ ^]^ and evolved/designed aldolases.^[^ ^131^, ^132^ ^]^ Zhou and co‐workers demonstrated that an artificial enzyme with a pyrrolidine‐based catalytic center assembled through disulfide formation with a cysteine residue could form enamine intermediates from cyclic ketones to enable a Michael addition to nitrostyrene derivatives (Figure 4b).^[^ ^133^ ^]^ Optimization of the position of pyrrolidine moiety and directed evolution of the surrounding residues in the LmrR scaffold afforded a variant with excellent stereoselectivity for this transformation. The streptavidin–biotin technology has also been applied for enamine catalysis to conduct an aldol reaction between acetone and nitrobenzaldehyde; however, poor stereoselectivity was obtained despite mutagenesis of the streptavidin scaffold being conducted.^[^ ^134^ ^]^ This is made more challenging by the highly electrophilic aldehyde substrate used, which affords a significant racemic background reaction.

### 4.2 Other Modes of Catalysis

By now, mechanisms in amine catalysis have been extensively elaborated for synthetic chemistry, and the development of new catalysts and mechanistic strategies has largely moved on to other activation modes, which are now also being explored for the development of artificial enzymes. Nucleophilic catalysis by the ncAA *N*‐methyl histidine (NMH) has been demonstrated by the group of Green, initially for ester hydrolysis,^[^ ^135^ ^]^ but now also for a Morita–Baylis–Hillman reaction to create a so‐called MBHase (Figure 4c).^[^ ^136^ ^]^ Early work by Reetz showed modest catalytic activities of various albumins in the MBH reaction, with low corresponding enantioselectivities.^[^ ^137^ ^]^ Thereafter, a computational designed MBHase with a theozyme envisaged with a catalytic histidine was produced by Baker and co‐workers; however, extremely poor activities were obtained until almost 10 years later when an extensive directed evolution campaign introducing 24 additional mutations over 14 rounds by Green to produce BH32.8.^[^ ^138^, ^139^ ^]^ Substitution of the catalytic histidine with NMH gave a large loss in activity, but a further eight rounds of directed evolution introducing 23 mutations gave a variant with a higher activity than the previously evolved MBHase which now gained a preference for NMH over histidine. This NMH‐>His substitution gave only a fourfold loss of activity, suggesting that the resulting variant with only natural amino acids might be a more practical biocatalyst.

Ward and Matile recently developed an antimony‐containing artificial enzyme based on the streptavidin–biotin technology which activates a quinoline substrate for transfer hydrogenation via σ‐hole interactions (Figure 4d).^[^ ^140^ ^]^ This generic activation mode may enable an array of new biocatalytic transformations by artificial enzymes containing antimony or other pnictogens/chalcogens.^[^ ^141^ ^]^ Boronic acid catalysis is an emerging methodology with a variety of unique mechanisms, but where stereoselectivity has been historically challenging.^[^ ^142^ ^]^ Use of the boronic‐acid containing ncAA *para*‐boronphenylalanine (pBoF) as a catalytic residue in the LmrR scaffold has recently created artificial enzymes for enantioselective catalysis via mechanisms unique to boron (Figure 4e).^[^ ^143^, ^144^ ^]^ The Roelfes group demonstrated an oxime‐formation reaction of alpha‐hydroxyketones was catalyzed via binding of a transient hemiaminal intermediate to the boron center, and directed evolution identified a triple mutant with hugely increased stereoselectivity in a kinetic resolution process. Crystallography and mutagenesis experiments suggested that a neighboring asparagine residue was essential for enabling efficient and selective catalysis through synergy with the unnatural pBoF sidechain. In another study from the group of Xiang, a Friedel–Crafts alkylation reaction of chalcone derivatives bearing a phenol moiety (crucial for binding to boron) was elaborated. Each study identified a different optimal position for pBoF incorporation, and different mutational substitutions in the subsequent directed evolution campaigns, demonstrating that fine‐tuning of the protein microenvironment around the pBoF can enable divergent reactivity profiles. Most recently, Roelfes, Longwitz, and co‐workers employed the RamR scaffold protein to produce an artificial enzyme with a pBoF catalytic residue which performs a hydrolytic kinetic resolution of alpha‐hydroxythioesters where a proximal lysine residue was identified for its potential direct involvement in the catalytic cycle.^[^ ^145^ ^]^ Artificial enzymes which operate via hydrogen‐bonding interactions are yet to be described, yet this mode of catalysis is common among natural enzymes and also in small‐molecule organocatalysis.^[^ ^146^, ^147^ ^]^ A recent study from Green showed that BH32.8 has promiscuous and evolvable activity for nucleophilic aromatic substitution reactions, likely powered a key arginine residue reminiscent of reactivity demonstrated with hydrogen bond donor catalysts, which may pave the way toward further new‐to‐nature biocatalysis powered by hydrogen bonding.^[^ ^148^, ^149^ ^]^

## 5 Photocatalysis and Synergy

Photocatalysis and metallaphotoredox catalysis are very active areas of research for the development of new synthetic methods enabled by light, as well as new transition metal mediated reaction pathways triggered by photoactivation, and significant progress is being made in improving the industrial applicability of these processes.^[^ ^150^, ^151^, ^152^, ^153^ ^]^ Nature also uses light to power metabolic enzymes with photoactive cofactors, and the merger of photo‐ and biocatalysis is finding increasing application for organic synthesis.^[^ ^154^, ^155^ ^]^ In particular, flavin‐dependent ene‐reductases have proven a versatile platform for promiscuous light‐powered reactivity through direct cofactor excitation.^[^ ^156^ ^]^ However, the scope of photobiocatalytic reaction mechanisms accessible with natural enzyme building blocks does not compare with the diversity of those available with organic chemistry, thus the development of photobiocatalysis with unnatural photocatalytic components is a vibrant area of current research. Synthetic photocatalysts are being merged with protein scaffolds to afford photocatalytic artificial enzymes, as well as systems that operate via metallaphotoredox catalysis, and synthetic photocatalysts can also create radical species that synergize with natural enzymatic reactive intermediates to unlock new catalytic reaction pathways.

### 5.1 Photocatalytic Artificial Enzymes

**Figure 5:**
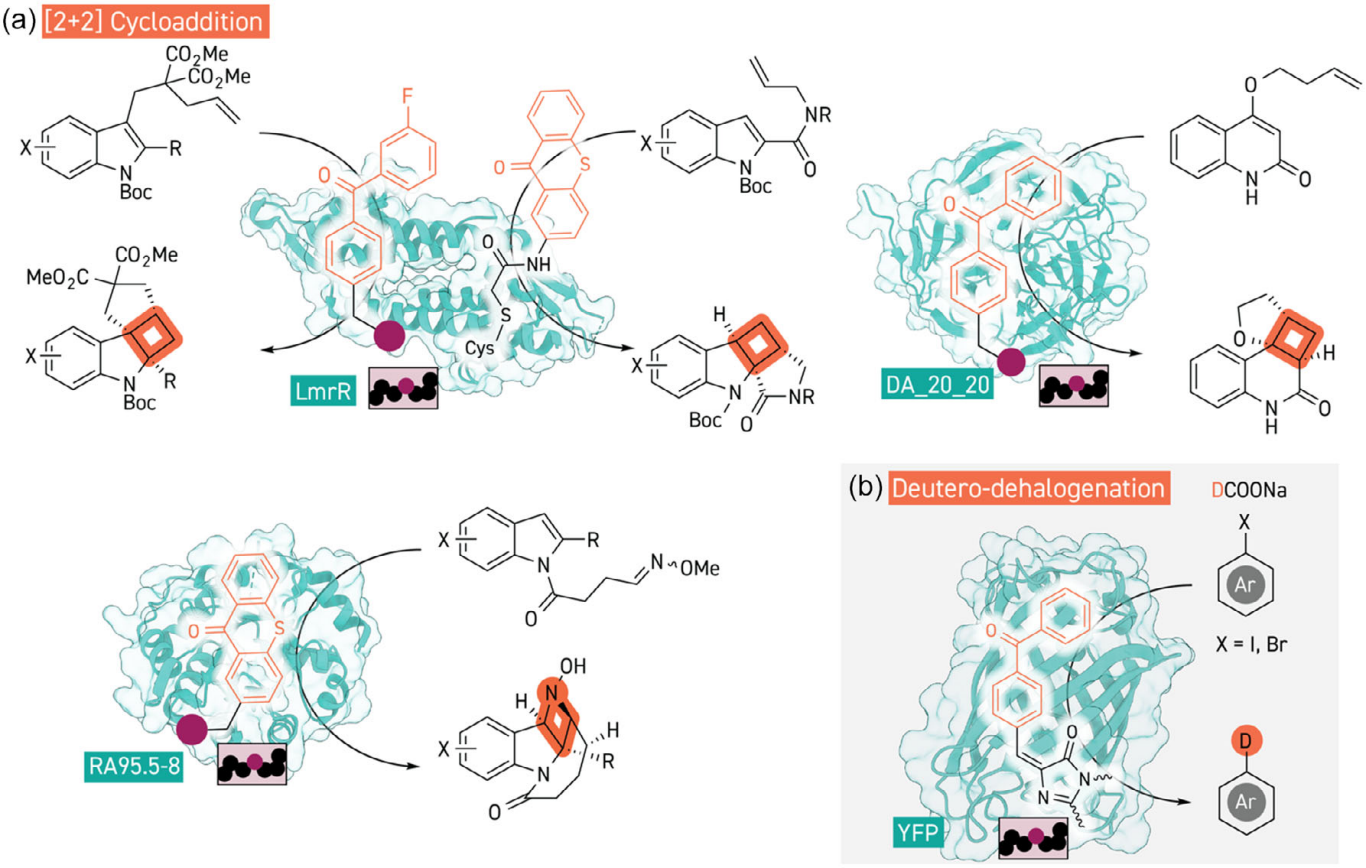
Photocatalytic artificial enzymes. a) [2 + 2] cycloadditions with artificial enzymes with genetically encoded or covalently attached benzophenone‐ and thioxanthone‐based photosensitizers to form cyclobutane and *N*‐methoxyazetidine products (PDB 3F8B, 7ZP5, and 8XYN). b) Deuterative dehalogenation of aryl halides in the presence of deuterium labeled formate catalyzed by YFP variant with pBpF genetically encoded into the chromophore motif(PDB 5YR2).

Organic photocatalysts based on the benzophenone and thioxanthone motifs are broadly applied in a range of synthetic processes.^[^ ^157^ ^]^ Bach demonstrated that supramolecular approach of catalyst desymmetrization by modification of one face of the thioxanthone core with a lactam H‐bonding recognition motif enables enantioselective inter‐ and intramolecular [2 + 2]‐cycloadditions with the recognized 2‐pyridone or quinolone substrates.^[^ ^158^, ^159^ ^]^ Inspired by these results, both Green and a collaborative group of Wu, Zhong and Chen simultaneously explored the utilization of benzophenone‐based ncAAs (*para*‐benzoylphenylalanine, pBpF) to create artificial photocatalytic enzymes for intramolecular [2 + 2] cycloadditions with quinoline and indole substrates, using scaffold proteins of a computationally designed Diels‐Alderase (DA2020) and LmrR, respectively (**Figure** **5**a).^[^ ^160^, ^161^ ^]^ Importantly, these artificial photoenzymes did not exhibit significant oxygen sensitivity which is a common issue for reactions that require triplet processes due to reactive oxygen species generation. Both studies conducted optimization of their artificial enzymes by directed evolution, with Wu's study finding that modification of pBpF to include a fluoro‐moiety gave a mild improvement in reaction stereoselectivity. Wu's group went further to demonstrate that the use of a bioconjugation approach (instead of an ncAA‐based approach) by cysteine modification with an iodoacetamide linker enabled subsequent chemical and genetic optimization processes, and identified superior performance with a thioxanthone based catalytic center.^[^ ^162^ ^]^ Most recently, Xiang and co‐workers engineered a new OTS for genetic encoding of a thioxanthone‐based ncAA which they incorporated into the hydrophobic pocket of designer retro‐aldolase RA95.5‐8.^[^ ^163^ ^]^ Instead of evaluating dialkenes for intramolecular cyclobutane formation, they exploited the same [2 + 2] mechanism with oxime containing indole derivatives to form cyclic *N*‐methyoxyazetidine products, with similar reactions previously being demonstrated in a racemic fashion by You and Schindler.^[^ ^164^, ^165^ ^]^ This new ncAA afforded a 20‐fold increase in turnover frequency compared to the benzophenone containing variant, and five rounds of directed evolution identified a variant with very high enantioselectivity as well as improved kinetic parameters and reaction yield. Various bioconjugation approaches with organic photocatalysts have been employed to produce artificial enzymes for sulfoxidation reactions; however, enantioselectivity could not be achieved to date, while enantioselective catalysis of this reaction with Baeyer–Villiger mono‐oxygenases is well explored.^[^ ^166^, ^167^, ^168^, ^169^ ^]^ Jiangyun Wang and co‐workers have shown that pBpF is accepted into the matured chromophore of yellow fluorescent protein in place of the canonical tyrosine, where it enables photocatalytic deuterative dehalogenation of aryl halides or can act as a photo reductant for radical SAM enzyme partners (Figure 5b).^[^ ^170^, ^171^ ^]^

Photosensitizers based on metal complexes, particularly of iridium and ruthenium, are extremely widely used in synthesis. Lewis has demonstrated the use of bioconjugation to incorporate such catalysts into the hydrophobic pocket of prolyl oligopeptidase from *Pyrococcus furiosus*, where a chemogenetic strategy could be used to tune photophysical properties.^[^ ^172^, ^173^ ^]^ Cycloadditions, predominantly with a [2 + 2] mechanism, were explored; however, the generation of a significant enantiomeric excess proved challenging, even when using only one photocatalyst enantiomer to construct the synthetic cofactor. Constitution of ruthenium and cobalt complexes into protein scaffolds has recently been used as a strategy to construct artificial enzymes for photocatalytic water splitting.^[^ ^174^, ^175^ ^]^ While in the case of the ruthenium cofactor interaction with the protein scaffold boosted activity, with the cobalt cofactor performance worsened. However, these systems may become promising with significant genetic optimization. Zeymer has recently shown that a de novo designed lanthanide binding protein exhibits activity for photocatalytic 1,2‐diol cleavage of hydrobenzoin substrates.^[^ ^176^, ^177^ ^]^ Mutagenesis of active‐site tryptophan residues improved photostability of this artificial photoenzyme by obviating some of the photodamage, but the broad active‐site cavity did not permit effective kinetic resolution of racemic substrates. The authors also identified that reconstitution of a natural lanthanide binding enzyme with cerium produces an active enzyme for this transformation, albeit with slight lower conversion than their designed protein. Most recently, the same group demonstrated that the pyrroloquinoline quinone cofactor which is found bound to lanthanide and calcium ions in the active sites of sugar and alcohol dehydrogenases also possesses photocatalytic properties, identifying several such enzymes that could perform enantioselective radical cyclization reactions.^[^ ^178^ ^]^ Further developments in promiscuous photobiocatalytic reactivity can be expected from these intriguing natural enzymes and engineered variants thereof.

### 5.2 Metallaphotoredox Biocatalysis

**Figure 6:**
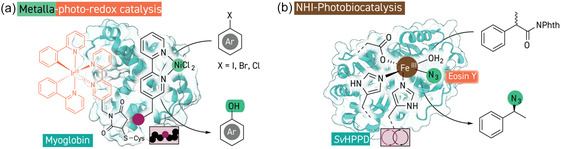
Merging photocatalysis and metalloredox catalysis in enzymes. a) An artificial enzyme for metallaphotoredox catalysis which employs a covalently linked iridium‐based photosensitizer together with a nickel(II) center bound at a genetically encoded chelating amino acid and can catalyze C—O couplings between aryl halides and water (PDB 6M8F). b) Photocatalytic formation of carbon radicals is merged with azide transfer reactivity of nonheme iron enzymes to give chiral azide products in an enantioconvergent manner (PDB 1T47).

Pioneering studies from Harry Gray demonstrated that electrons could tunnel over distances of 15 Å from ruthenium complexes modified on the surface of cytochrome c to the heme‐containing active site.^[^ ^179^, ^180^ ^]^ Such electron transfer processes are involved in the natural modulation of metalloenzyme reactivity and lie are the core of the field of metallaphotoredox catalysis, setting the stage for exploiting of this phenomenon to design enzymatic reactivity.^[^ ^150^ ^]^ Jiangyun Wang's group demonstrated that further modification YFP protein that contains the photoactive ncAA pBpF in its chromophore with a nickel tri‐ or bipyridine complex by iodoacetamide bioconjugation enables either CO_2_ reduction or aryl halide hydroxylation via metallaphotoredox mechanisms involving long‐range electron transfer.^[^ ^181^, ^182^ ^]^ With a strategy based on bioconjugation of an iridium‐based photosensitizer and incorporation of a nickel center with the use of a metal‐binding ncAA with apomyoglobin as scaffold protein, Song and Lee also recently elaborated an artificial enzyme for dehalogenation and hydroxylation of aryl halides (**Figure** **6**a).^[^ ^183^ ^]^ As with the studies from Jiangyun Wang, they found that optimization of the distance between the two unnatural catalytic components by strategic placement of the cysteine residue used for bioconjugation was an effective way to optimize the activity and selectivity of these dual‐catalytic systems.

Metallaphotoredox biocatalysis can also be achieved through synergy of exogenous photocatalysts with nonheme iron enzymes, which canonically hydroxylate or halogenate organic substrates.^[^ ^184^ ^]^ Xiongyi Huang and co‐workers demonstrated that (4‐hydroxyphenyl)pyruvate dioxygenase from *Streptomyces avermitilis* could mediate C—H azidation reactivity via an unnatural Fe^II^/Fe^III^ radical relay pathway triggered by homolytic cleavage of a substrate N—F bond (Figure6b).^[^ ^185^ ^]^ Also exploiting an Fe^II^/Fe^III^ cycle in the same enzyme, Xiongyi Huang has now demonstrated that azidation of photocatalytically generated radicals can also be achieved.^[^ ^186^ ^]^ As with the previously discussed thiamin reactivity, *N*‐(acyloxy)phthalimide radical precursors were activated by the Eosin Y photocatalyst to give benzylic or aliphatic radical species to which the azide was transferred from the iron center inside the enzyme. While racemic radical precursors were employed, the enantiomeric ratio of this starting material did not change over the reaction course, demonstrating enantioconvergent reactivity in the chiral environment of the enzyme. Contemporaneous work from Yang demonstrated the same reactivity instead with metapyrocatechase from *Pseudomonas putida* obtaining the opposite product enantiomer from Xiongyi Huang's study.^[^ ^187^ ^]^ Both groups found that replacing azide with thiocyanide enabled thiocyanation reactivity, and Yang further demonstrated a variety of functional‐group interconversions through follow‐up chemistry on the azide and thiocyanide products.

### 5.3 Synthetic Photocatalysts Synergizing with Natural Enzymes

Besides the concept of combining photocatalysts with transition metal complexes for metallaphotoredox catalysis, the combination of photocatalysts with organocatalysts has also proven extremely powerful for elucidating new mechanistic manifolds.^[^ ^188^ ^]^ Correspondingly, the exploration of promiscuous biocatalytic activities through the combination photocatalysts with enzymes featuring flavin, pyridoxal phosphate (PLP), or thiamine cofactors has proven a fruitful avenue to identify new photobiocatalytic possibilities.

**Figure 7:**
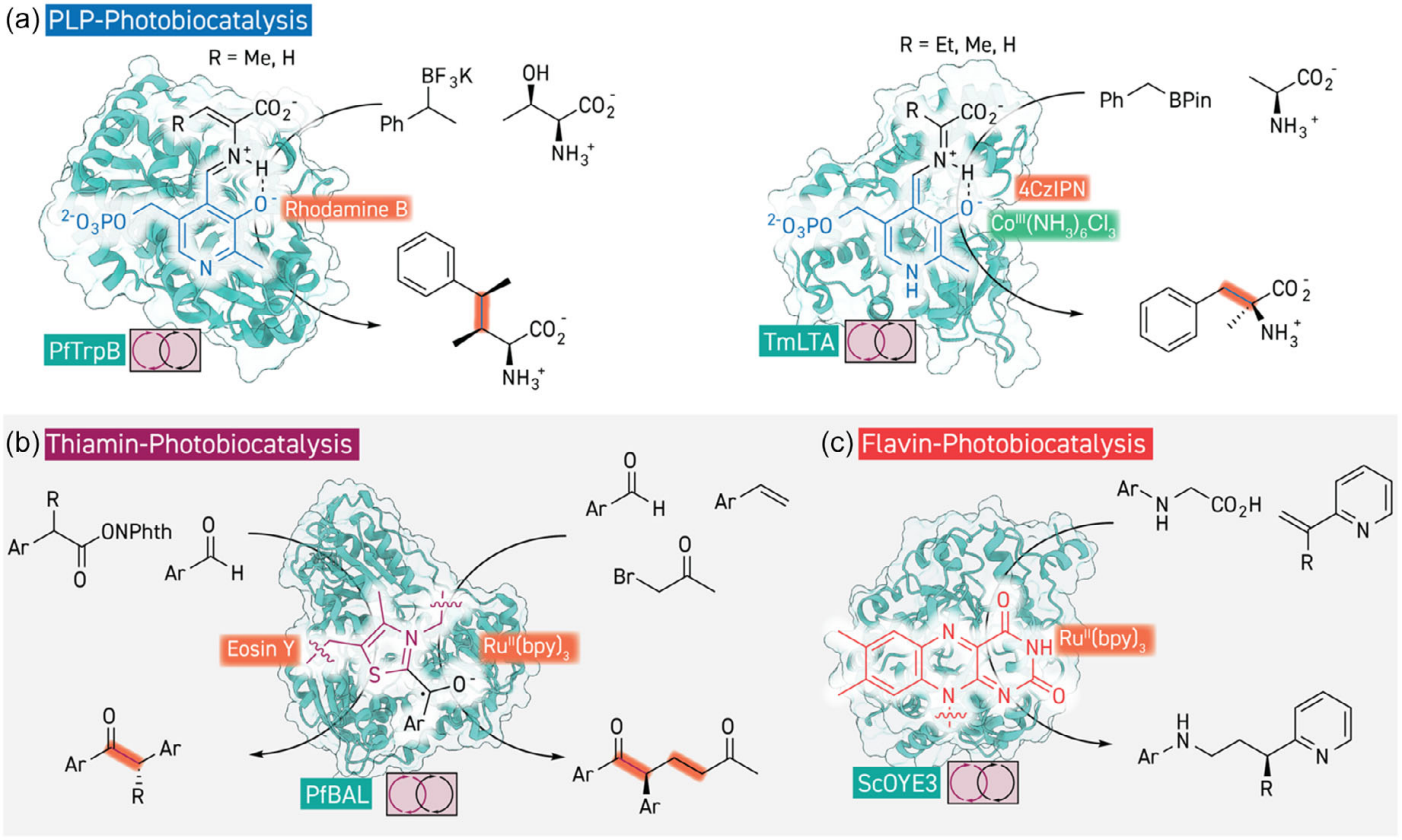
Merging enzymes with chemical photocatalysts for new radical reactivity. a) Combination of PLP‐dependent enzymes with photocatalytically generated radical species facilitates the synthesis of ncAA products (PDB 5DVZ and 1LW4). b) Photocatalysts facilitate formation of a noncanonical ketyl‐radical intermediate in the active site of thiamin‐dependent enzymes which allows radical acylation, also via triple radical sorting (PDB 2AG0). c) Decarboxylative radical formation mediated by a ruthenium photocatalyst in the active site of a flavin‐dependent ene‐reductase affords radical addition to vinyl pyridine substrates (PDB 5V4V).

PLP‐dependent enzymes feature heavily in amino acid biosynthesis, and expansion of the substrate scopes through protein engineering of, for example, tryptophan synthase, threonine aldolase, and tyrosine phenol lyase has facilitated the synthesis of a broad variety ncAAs via these enzymes’ associated canonical, two‐electron, mechanisms.^[^ ^189^ ^]^ In contrast to the applications of ncAAs to create enzymes, here we see examples of enzymes creating ncAAs, which could present a convenient and sustainable route to these building blocks that find broad application in chemical biology, as well as drugs. One particular breakthrough was the engineering of so‐called “stand‐alone” tryptophan synthase beta‐subunits (TrpB) which is responsible for connection of the indole moiety of the tryptophan sidechain but normally requires the alpha‐subunit for allosteric activation.^[^ ^190^ ^]^ These “stand‐alone” variants could be further engineered for synthesis of a broad range of ncAAs exploiting TrpBs natural mechanism with both aromatic and nonaromatic nucleophilic substrates. Synergy with photocatalysts for in situ radical generation has opened pathways for the demonstration of one‐electron mechanisms in these enzymes, which, in turn, allows new disconnections in amino acid retrobiosynthesis. In 2023, Yang and colleagues demonstrated that the aminoacrylate intermediate formed by condensation of serine at the PLP cofactor of a stand‐alone TrpB variant could undergo one‐electron C—C bond forming reactions with photocatalytically generated carbon‐centered radical species.^[^ ^191^ ^]^ Using Rhodamine‐B as photocatalyst together with benzylic BF_3_K‐salts as radical precursor, serine could be converted into homophenylalanine derivatives by this synergistic photobiocatalytic system (**Figure** **7**a). Protein engineering identified a single E104G mutant, which removes a sidechain that is essential for the enzyme's native reactivity^[^ ^192^ ^]^ that could invert the stereochemistry at the alpha‐carbon, affording access to amino acid products with the unnatural d‐configuration. Aliphatic BF_3_K‐salts could also be employed as substrates, as well as secondary BF_3_K‐salts which, together with threonine as partner substrate, afforded products with up to three contiguous stereocenters with good diastereomeric purity. A follow‐up study performed directed evolution to improve enantioselectivity of the reaction when conducted with benzylic boron pinacol ester radical precursors demonstrated that the mutations introduced abrogated unexpected racemase activity present in the starting variant.^[^ ^193^ ^]^ More recently, radical mechanisms in PLP‐dependent enzymes via synergistic photocatalysis were expanded to threonine aldolase, another PLP‐dependent enzyme which forms a quinoid intermediate via either retro‐aldol or deprotonation processes which is nucleophilic at the amino acid alpha‐carbon.^[^ ^194^ ^]^ A synergistic photobiocatalytic cycle was constructed with the threonine aldolase from *Thermotoga maritima* using BF_3_K‐salts or boron pinacol esters as radical precursor, iridium or cyanoarene photocatalyst and stoichiometric Co(III) oxidant which achieved radical C—C coupling.^[^ ^195^ ^]^ This pathway affords phenylalanine derivatives, rather than the homophenylalanine analogues produced by the TrpB‐facilitated reaction, starting from simple glycine (Figure 7a). Moreover, alanine and 2‐aminobutryic acid could also be employed as substrates to afford ncAAs with tetrasubstitution at the alpha‐carbon featuring methyl and ethyl groups, respectively. Pyridinium salts were also used as radical precursors for synergistic photobiocatalysis with threonine aldolase by both Hyster and Yang, with the latter identifying that with these substrates the photocatalyst could be obviated.^[^ ^196^, ^197^ ^]^ Electron paramagnetic resonance spectroscopy and luminescence decay measurements indicated that the external aldimine intermediate formed by condensation of threonine aldolase with alanine or glycine can itself operate as a photosensitizer. Notably, while biomimetic chemical catalysis employed PLP‐inspired carbonyl catalysts is a growing area,^[^ ^198^ ^]^ the PLP‐enzyme synergistic photocatalytic transformations described above have not been demonstrated with any other catalyst class, demonstrating that innovation in biocatalysis can go beyond chemomimicry and into the elucidation of completely novel reactions.

Thiamin‐dependent enzymes, whose cofactor typically condenses with aldehyde substrates to form the Breslow enolate intermediate, have also been recently demonstrated as amenable to radical mechanisms in combination with radical precursors and photocatalysts. In 2019, Ohmiya, Nagao, and co‐workers demonstrated that small‐molecule *N*‐heterocyclic carbene catalysts could enable a radical acylation of aldehydes to form the corresponding racemic ketones by employing *N*‐(acyloxy)phthalimide radical precursors.^[^ ^199^ ^]^ Employing benzaldehyde lyase from *Pseudomonas fluorescens* and Eosin Y used to generate radicals from the same precursor class, Xiaoqiang Huang's group enabled this reactivity in a biocatalytic fashion, affording enantioselective acylation reaction from aromatic aldehydes, overriding the canonical benzoin formation reactivity (Figure 7b).^[^ ^200^ ^]^ Interestingly, the small molecule equivalent of this reaction does not require photocatalyst or irradiation but rather relies on radical formation by a proposed single electron transfer from the Breslow enolate. In the biocatalytic system, electron paramagnetic resonance spectroscopy and computational studies suggested that the Eosin Y photocatalyst is responsible both for generating radicals from the *N*‐(acyloxy)phthalimide substrates as well as direct excitation of the Breslow intermediate to form a ketyl‐radical. Expanding on this work, a three component radical sorting reaction was realized using photocatalytic ruthenium complexes which perform a radical addition of acyl‐bromides to styrenes, a reactivity previously also demonstrated racemically by Ohmiya, Nagao, and co‐workers with a single NHC catalyst system.^[^ ^201^, ^202^ ^]^ The resulting benzylic radical product undergoes coupling with the ketyl‐radical species formed at the thiamin cofactor in a stereoselective fashion. Each of the three reaction substrates could be varied to a certain degree giving modular access to a series of products with most being formed with almost perfect stereoselectivity. Fasan has also demonstrated benzylic C—H radical acylation with the same enzyme platform, using *N*‐fluorobenzamide H‐atom abstraction reagents to form benzylic radicals which couple with the thiamine ketyl‐radical.^[^ ^203^ ^]^ Exploiting the reversibility of the benzoin condensation reaction, the benzaldehyde substrates could be replaced with benzoin, supporting involvement of the Breslow intermediate in the reaction cycle. Together with radical trapping and kinetic isotope effect experiments, this study suggested a mechanism in line with other proposals on thiamine‐based radical photobiocatalysis.

In recent years, radical reactivity with flavin dependent enzymes have been extensively elaborated, principally by Hyster and co‐workers, where direct photoexcitation of a charge‐transfer complex between substrate and the FMN‐cofactor facilitates one‐electron mechanisms.^[^ ^204^ ^]^ Synergistic combination of these enzymes with photosensitizer dyes, however, has expanded the range of radical species which can be formed, and thus the resulting product scope. For example, decarboxylative radical formation from *N*‐arylated glycine derivatives facilitated by a ruthenium photocatalyst, which undergo addition to vinyl‐pyridine substrates in the active site of OYE3 from *Saccharomyces cerevisiae* (Figure 7c).^[^ ^205^ ^]^ The product stereocenter is formed by hydrogen atom transfer from the FMN hydroquinone which is then regenerated from its semiquinone form by single‐electron transfer from the ruthenium photocatalyst. Fluorescence quenching experiments between the enzyme and ruthenium complex indicated static quenching by tight association between photocatalyst and active site, with a low micromolar dissociation constant being calculated. The synthetic utility of this system was demonstrated by application to the chemoenzymatic synthesis of an antihuman cytomegalovirus compound and a gamma‐lactam. The same ruthenium photocatalyst could also be employed to generate nitrogen‐centered radicals from hydroxamic esters which undergo intramolecular 6‐endo‐trig hydroamination in the active site of YqjM, an ene‐reductase from *Bacillus subtilis*, with subsequent enantioselective hydrogen atom transfer from the FMN hydroquinone forming the corresponding delta‐lactam products.^[^ ^206^ ^]^ A directed evolution campaign identified enantiodivergent YqjM variants for this reactivity, and switching the enzyme to GluER from *Gluconobacter oxydans* enabled the same reactivity but in an intermolecular fashion. Xiaoqiang Huang and colleagues recently demonstrated diastereo‐ and enantio‐selective synthesis of lactones from gamma,delta‐unsaturated carboxylic acids through synergy between GluER and rhodamine 6 G photocatalyst.^[^ ^207^ ^]^ They hypothesized that direct single‐electron excitation of the cofactor by the photocatalyst facilitates radical formation at the substrate double bond, whereupon intramolecular cyclization and enantioselective hydrogen atom transfer can complete product formation. Finally, Yajie Wang has recently shown that malonitrile can undergo radical addition to alpha‐methyl styrene derivatives through synergistic action between fluorescein photocatalysts and ene‐reductases.^[^ ^208^ ^]^ Screening a small library of ene‐reductase variants identified an enantiocomplementary pair, while computational and experimental mechanistic studies suggested that fluorescein's role is to cycle the FMN cofactor between its oxidized and semiquinone forms.

The group of Melchiorre has recently also demonstrated that radical photocatalytic processes can expand the reaction organocatalytic reaction pathways possible with 2‐deoxyribose 5‐phosphate aldolase, where direct photoexcitation of an enzyme‐iminium intermediate facilitated the reaction and exogenous photocatalyst was not required.^[^ ^209^ ^]^ Looking forward, many more classes of enzymes will likely have their respective mechanistic pathways expanded through the use of photocatalytic processes, with or without the help of small‐molecule photocatalysts.

## 6 The Future of Enzyme Design

Throughout this review, which covers predominantly progress realized in the last few years, we have seen how an expanding the enzyme designer's palette is driving rapid expansion of biocatalysis's possibilities. From designing enzymes with unnatural catalytic metallic or organic machinery at their core, to synergizing photocatalytic radical generation with enzyme mechanisms, the addition of unnatural chemical components to biocatalysis is a powerful recipe for innovation. Ultimately these efforts add to the creation of chemomimetic reactivity in enzymes, increasing their potential for the replacement of chemocatalytic methods as well as incorporation into novel unnatural biosynthetic pathways.^[^ ^16^, ^73^, ^210^, ^211^ ^]^ More exciting still, in some cases these approaches allow expansion beyond reaction pathways that have been achieved chemically, providing a new unique advantage for biocatalysis.

**Figure 8:**
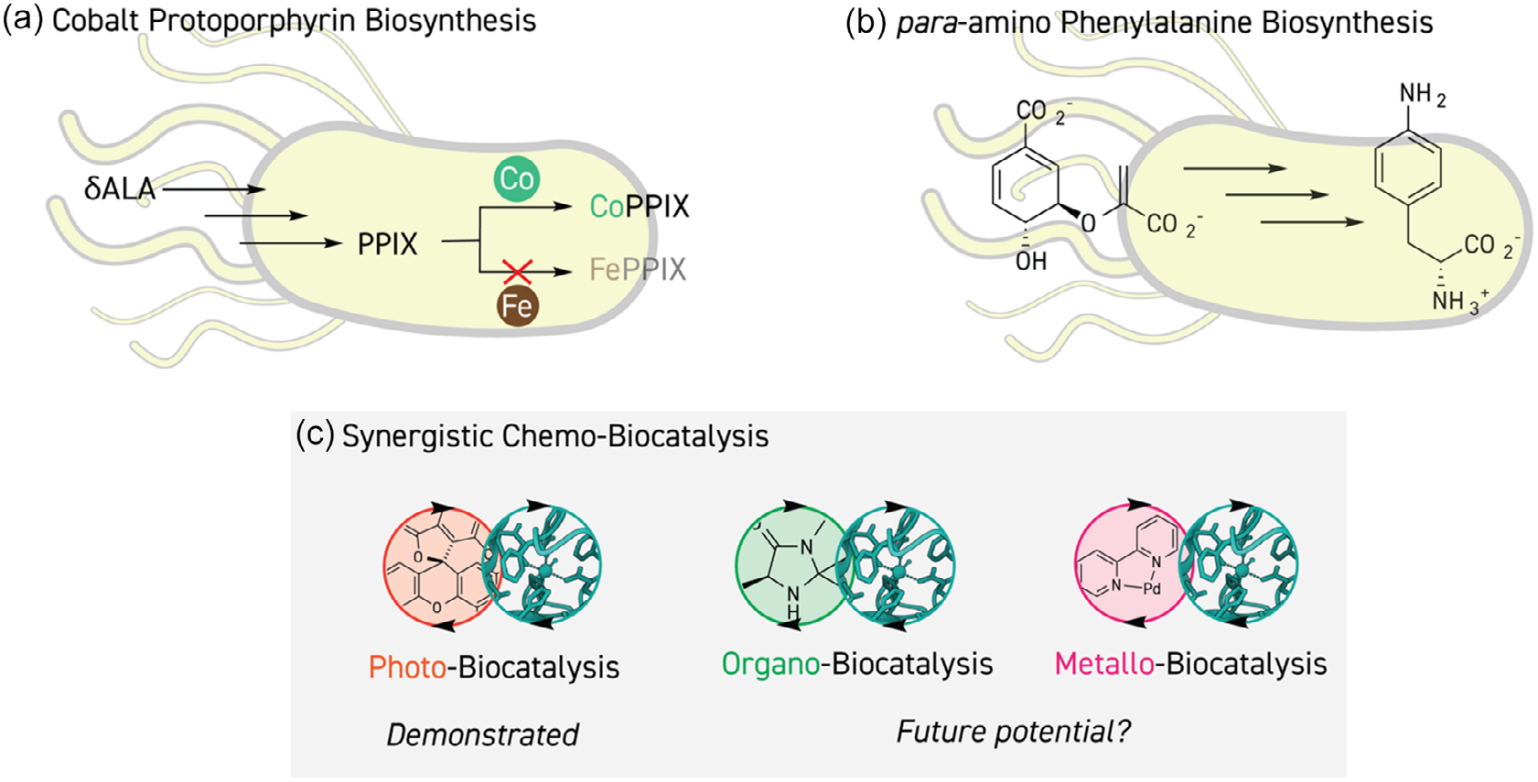
Future considerations for the use of an expanded palette for enzyme design. a) Biosynthesis of an unnatural cobalt‐porphyrin in *E. coli* by supplementation of the medium with cobalt, reducing heme production. b) Biosynthesis of the ncAA *para*‐amino phenylalanine by introduction of a new pathway in *E. coli*. c) New reaction manifolds for synergistic chemo‐biocatalysis, expanding on photo‐biocatalysis to include the merger of organocatalytic and metallic reactivities with enzyme mechanisms.

In many of the examples in this review, the resulting reactivity is not known to occur without the use of an expanded enzyme design palette, yet in some cases all‐natural biocatalytic alternatives do exist. This calls into question the usage of unnatural chemical species in the enzyme design since their application is not without its costs. In many cases, enzyme production is complicated which can hamper protein engineering efforts but, more importantly, the inclusion of unnatural components greatly increases the ultimate enzyme production costs. In situ biosynthesis of the required abiotic catalytic components is a potential solution to alleviate this burden and has already been demonstrated in the case of cobalt‐porphyrin where simple supplementation of the growth medium with cobalt chloride is sufficient to produce cobalt‐substituted heme proteins with high levels of cobalt‐loading (**Figure** **8**a).^[^ ^212^, ^213^ ^]^ *para*‐Amino phenylalanine, which has been used as catalytic ncAA in designed enzymes,^[^ ^113^, ^114^, ^115^ ^]^ can also be biosynthesized and genetically encoded in *E. coli* by incorporation of three enzymes from *S. venezuelae*, presenting a pathway to fully biosynthetic production of these artificial enzymes (Figure 8b).^[^ ^214^ ^]^

In the last section of this review, we saw how many new biocatalytic possibilities have been enabled through the synergistic combination of photocatalysts and enzymes. The approach of merging photocatalytic cycles with others is inspired by synergistic strategies in chemical catalysis.^[^ ^150^, ^188^ ^]^ However, synergistic catalyst combinations have also been realized, for example, with organocatalysis and transition metal catalysis which are both, at least in part, amenable to the reaction conditions required for enzymes, i.e., aqueous solvents.^[^ ^215^, ^216^, ^217^, ^218^ ^]^ Therefore, the combination of small‐molecule organocatalysts or transition metal catalysts with biocatalysts to merge their respective mechanisms in a synergy could well present a future direction for the expansion of bond forming strategies (Figure 8c).

Finally, it is noteworthy that perhaps most of the studies described herein employed intuitive design followed by directed evolution to achieve desired activity and selectivity for the target transformation. So far, computational design strategies do not feature heavily in this area, perhaps because nonproteogenic components can complicate computational workflows.^[^ ^219^ ^]^ However, the latest generation of enzyme design methodologies, relying on advances in AI‐powered protein design and structure prediction have greater success than their earlier counterparts, affording activities increased by several orders of magnitude compared to older, physics‐based, workflows.^[^ ^26^, ^27^, ^28^, ^29^ ^]^ In one recent study from the Ward and Baker groups, de novo design was used to create a binding site for a synthetic ruthenium cofactor to create an artificial enzyme for ring‐closing metathesis.^[^ ^220^ ^]^ This suggests that de novo design of enzymatic systems with unnatural components may now provide a future avenue to expand the repertoire of biocatalysis while minimizing or obviating the requirement for directed evolution.

## Conflict of Interest

The authors declare no conflict of interest.
